# Supplementary material for: Estimating heritability and genetic correlations from large health datasets in the absence of genetic data
Source: Nat Commun. 2019 Dec 3;10:5508. doi: 10.1038/s41467-019-13455-0 (PMC6890770; doi:10.1038/s41467-019-13455-0)
Supplement: Supplementary file 1 — Supplementary Information New [file 41467_2019_13455_MOESM1_ESM.pdf]

Supplementary Information

Estimating Heritability and Genetic Correlations from Large Health Datasets in the Absence of Genetic Data

Gengjie Jia [1]
Yu Li [2]
Hanxin Zhang [1,3]
Ishanu Chattopadhyay [1]
Anders Boeck Jensen [4]
David R. Blair [5]
Lea Davis [6]
Peter N. Robinson [7]
Torsten Dahlén [8]
Søren Brunak [9]
Mikael Benson [10]
Gustaf Edgren [8]
Nancy J. Cox [6]
Xin Gao [2]
Andrey Rzhetsky [1,3,11,*]

[1] Department of Medicine, and Institute of Genomics and Systems Biology, University of Chicago, Chicago, IL, 60637, US

[2] Computational Bioscience Research Center, Computer, Electrical and Mathematical Sciences and Engineering Division, King Abdullah University of Science and Technology (KAUST), Thuwal, 23955, Saudi Arabia

[3] Committee on Genomics, Genetics, and Systems Biology, University of Chicago, Chicago, IL, 60637, US

[4] Institute for Next Generation Healthcare, Department of Genetics and Genomic Sciences, Icahn School of Medicine at Mount Sinai, New York, NY, 10029, US

[5] Department of Pediatrics, University of California San Francisco, San Francisco, CA, 94158, US

[6] Division of Genetic Medicine, Vanderbilt University, Nashville, TN, 37232, US

[7] Jackson Laboratory for Genomic Medicine, Farmington CT, 06032, US

[8] Department of Medical Epidemiology and Biostatistics, Karolinska Institutet, Stockholm, SE-171 77, Sweden

[9] Novo Nordisk Foundation Center for Protein Research, Faculty of Health and Medical Sciences, University of Copenhagen, Copenhagen, 1017, Denmark

[10] Centre for Individualized Medicine, Department of Pediatrics, Linkoping University, Linkoping, 58183, Sweden

[11] Department of Human Genetics, University of Chicago, Chicago, IL, 60637, US

*andrey.rzhetsky@uchicago.edu

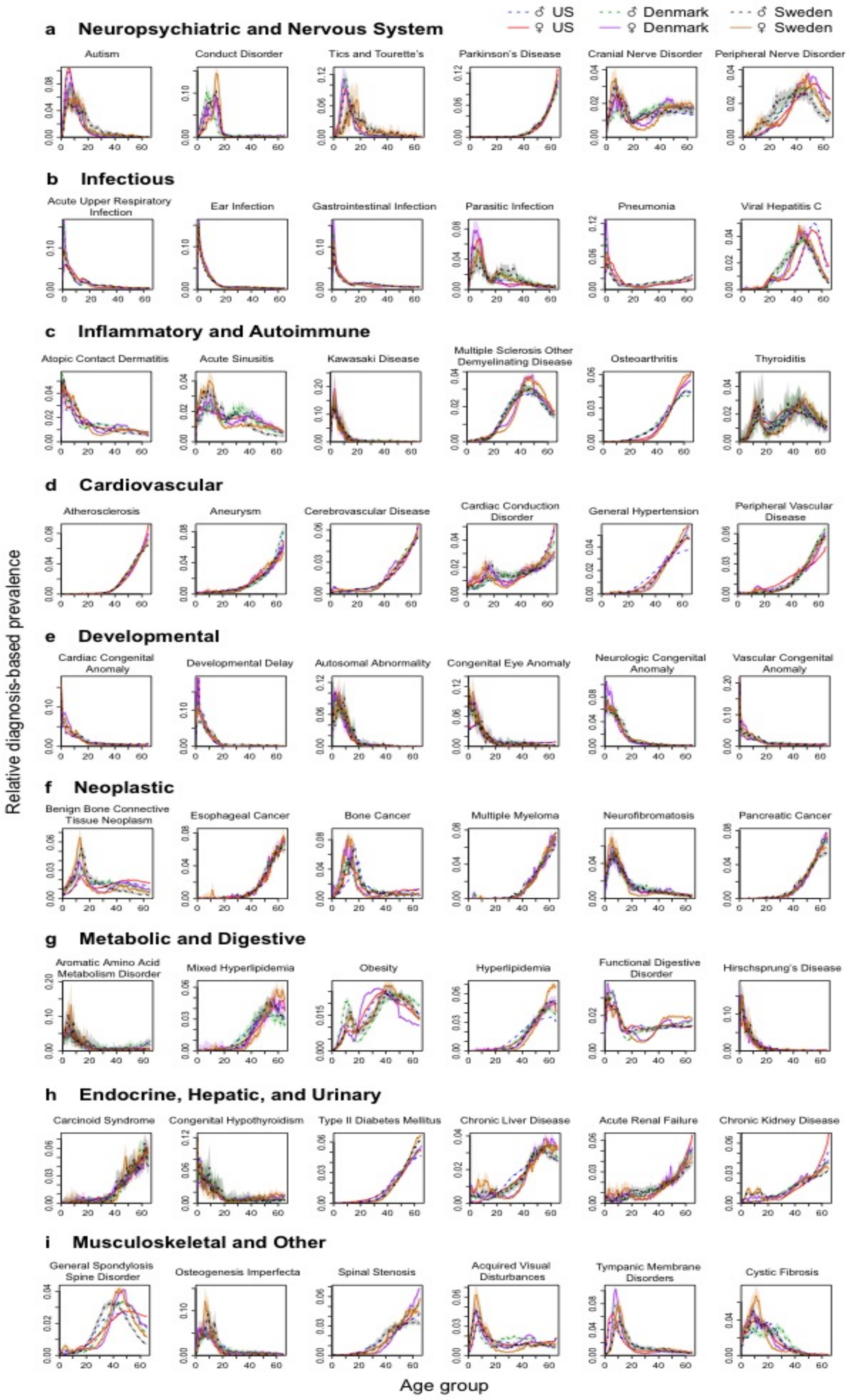

♂ US
♀ US
♂ Denmark
♀ Denmark
♂ Sweden
♀ Sweden
a Neuropsychiatric and Nervous System
Autism
Conduct Disorder
Tics and Tourette's
Parkinson's Disease
Cranial Nerve Disorder
Peripheral Nerve Disorder
b Infectious
Acute Upper Respiratory Infection
Ear Infection
Gastrointestinal Infection
Parasitic Infection
Pneumonia
Viral Hepatitis C
c Inflammatory and Autoimmune
Atopic Contact Dermatitis
Acute Sinusitis
Kawasaki Disease
Multiple Sclerosis Other Demyelinating Disease
Osteoarthritis
Thyroiditis
d Cardiovascular
Atherosclerosis
Aneurysm
Cerebrovascular Disease
Cardiac Conduction Disorder
General Hypertension
Peripheral Vascular Disease
e Developmental
Cardiac Congenital Anomaly
Developmental Delay
Autosomal Abnormality
Congenital Eye Anomaly
Neurologic Congenital Anomaly
Vascular Congenital Anomaly
f Neoplastic
Benign Bone Connective Tissue Neoplasm
Esophageal Cancer
Bone Cancer
Multiple Myeloma
Neurofibromatosis
Pancreatic Cancer
g Metabolic and Digestive
Aromatic Amino Acid Metabolism Disorder
Mixed Hyperlipidemia
Obesity
Hyperlipidemia
Functional Digestive Disorder
Hirschsprung's Disease
h Endocrine, Hepatic, and Urinary
Carcinoid Syndrome
Congenital Hypothyroidism
Type II Diabetes Mellitus
Chronic Liver Disease
Acute Renal Failure
Chronic Kidney Disease
i Musculoskeletal and Other
General Spondylosis Spine Disorder
Osteogenesis Imperfecta
Spinal Stenosis
Acquired Visual Disturbances
Tympanic Membrane Disorders
Cystic Fibrosis
Relative diagnosis-based prevalence
Age group

Supplementary Fig. 1 Disease curve examples which can be replicated using another independent cohort, the Swedish National Health Registry (related to Fig. 1a).

The Swedish dataset-specific curves (shown with black-dotted and brown solid lines) further validate the curve similarities seen between the US and Denmark datasets. For example, we successfully validated all of the aligned disease curves in the five clusters shown in Fig. 1c and included them here: Plate **a** shows autism, conduct disorder, tics and Tourette's, Parkinson's disease, and cranial nerve disorder; Plate **b** shows parasitic infection; Plate **c** shows osteoarthritis, acute sinusitis, thyroiditis, and Kawasaki disease; Plate **d** shows general hypertension and atherosclerosis; Plate **e** shows congenital eye anomaly; Plate **f** shows neurofibromatosis, pancreatic cancer, multiple myeloma, and esophageal cancer; Plate **g** shows hyperlipidemia; Plate **h** shows type II diabetes mellitus, and; Plate **i** shows tympanic membrane disorders and osteogenesis imperfecta. A curve's *X*-axis corresponds to the diagnosis assignment age, while the *Y*-axis shows the relative prevalence of each diagnosis in the corresponding age and sex group (the curves are further re-normalized so that area under the curve equals 1). Each curve is supplied with a 99 percent confidence interval.

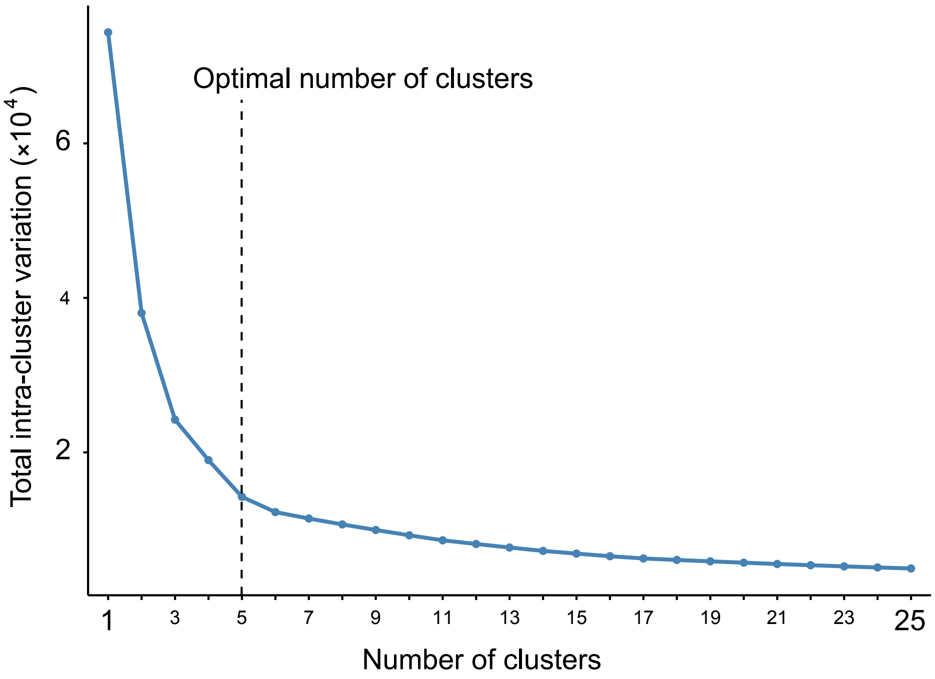

Supplementary Fig. 2 The elbow method determined the optimal number of shape-of-curve clusters (related to Fig. 1b).
Based on the matrix of dissimilarity measurements between all pairs of sex- and country-specific curves, we applied hierarchical clustering and calculated the total intra-cluster variation for cluster numbers ranging from 1 to 25. The *Y*-axis shows the total intra-cluster variation values and the *X*-axis corresponds to the total number of clusters. The five-cluster subdivision appears optimal, because it is where the decline of the variation value switches from fast to slow (*i.e.*, the elbow location).

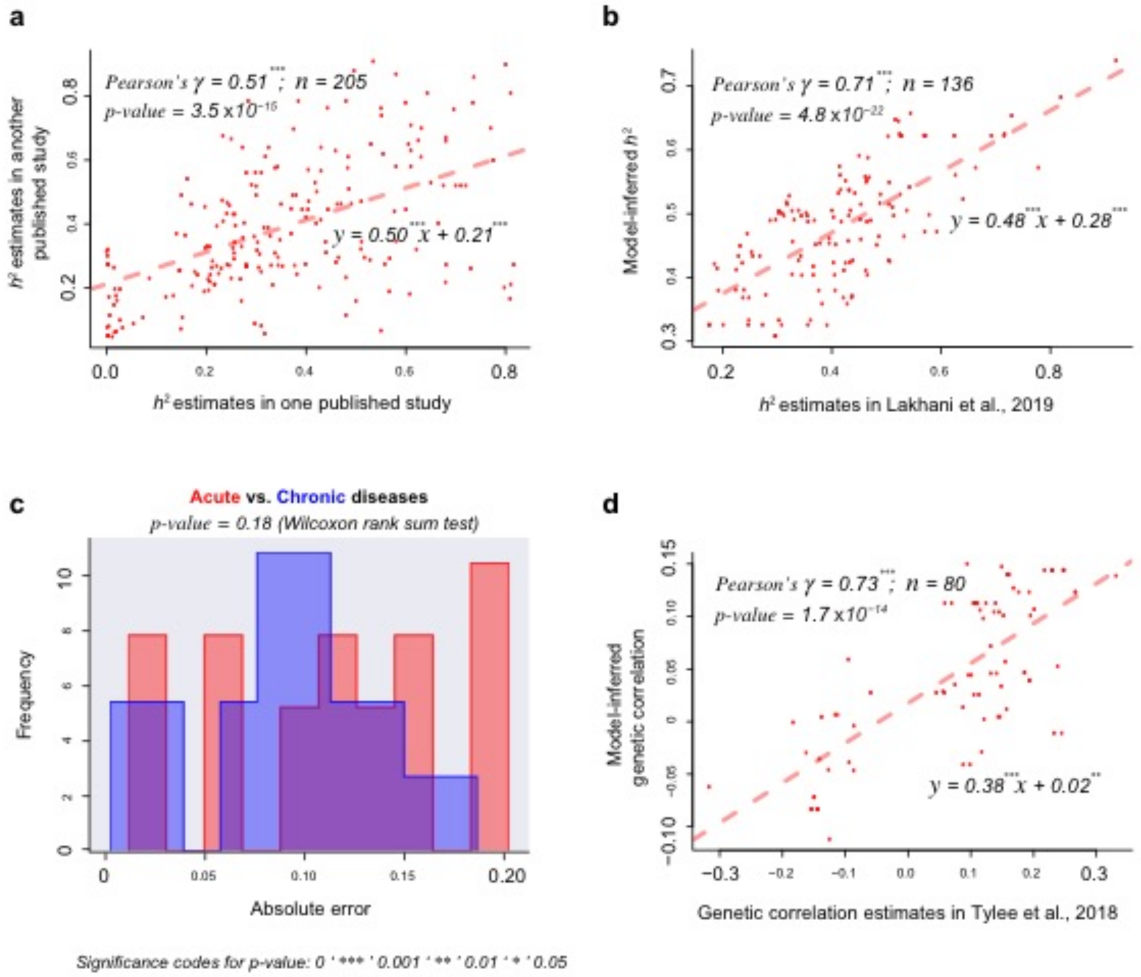

Supplementary Fig. 3 Correlation within legacy estimates of *h*² and between recently published estimates and our model predictions (related to Supplementary Data 3, 4, and 5).

a From 1146 published h^2 estimates, we selected 205 pairs that shared the same data type and modeling setup but were based on distinct populations or studies. Here, we display them as a scatterplot, where the X- and Y-coordinates of each point represent comparable h^2 estimates, obtained in pairs of independent studies. We computed a best-fitting regression line and Pearson's correlation coefficient (γ = 0.51, p-value was computed using Student's t test), serving as the baseline against which to benchmark. **b** We further brought in another test dataset from a very recently published study (CaTCH), which used EHR-inferred twin data. Our model predictions for the relevant data type are very consistent with theirs (γ = 0.71, see Supplementary Data 3 for comparison details), improving the correlation seen between legacy data (Plate **a**) by 0.2. **c** We revisit the comparison by only selecting the test results for a list of acute and chronic diseases, respectively (see Supplementary Data 4). We first compute absolute errors (*i.e.*, the absolute difference between model-inferred and published values), and then use the Wilcoxon rank sum test to determine whether the distribution of the errors seen in acute diseases is different from that of chronic diseases. This difference proves to be not

significant (p = 0.18), suggesting that the accuracy of model predictions for acute diseases is similar to that for chronic diseases. We therefore confirm that, as far as the proposed model is concerned, diseases, acute or chronic, are no different. **d** To validate our estimates of genetic correlations against an independent dataset, we found an additional dataset of genetic correlations and reserved it exclusively for testing purposes. Benchmarking against this test dataset that was generated based on genome-wide association studies and using linkage disequilibrium score (LDSC) regression, we compared our predictions for the same data type and mathematical method, again confirming a significantly high concordance (γ = 0.73, see Supplementary Data 5 for comparison details).

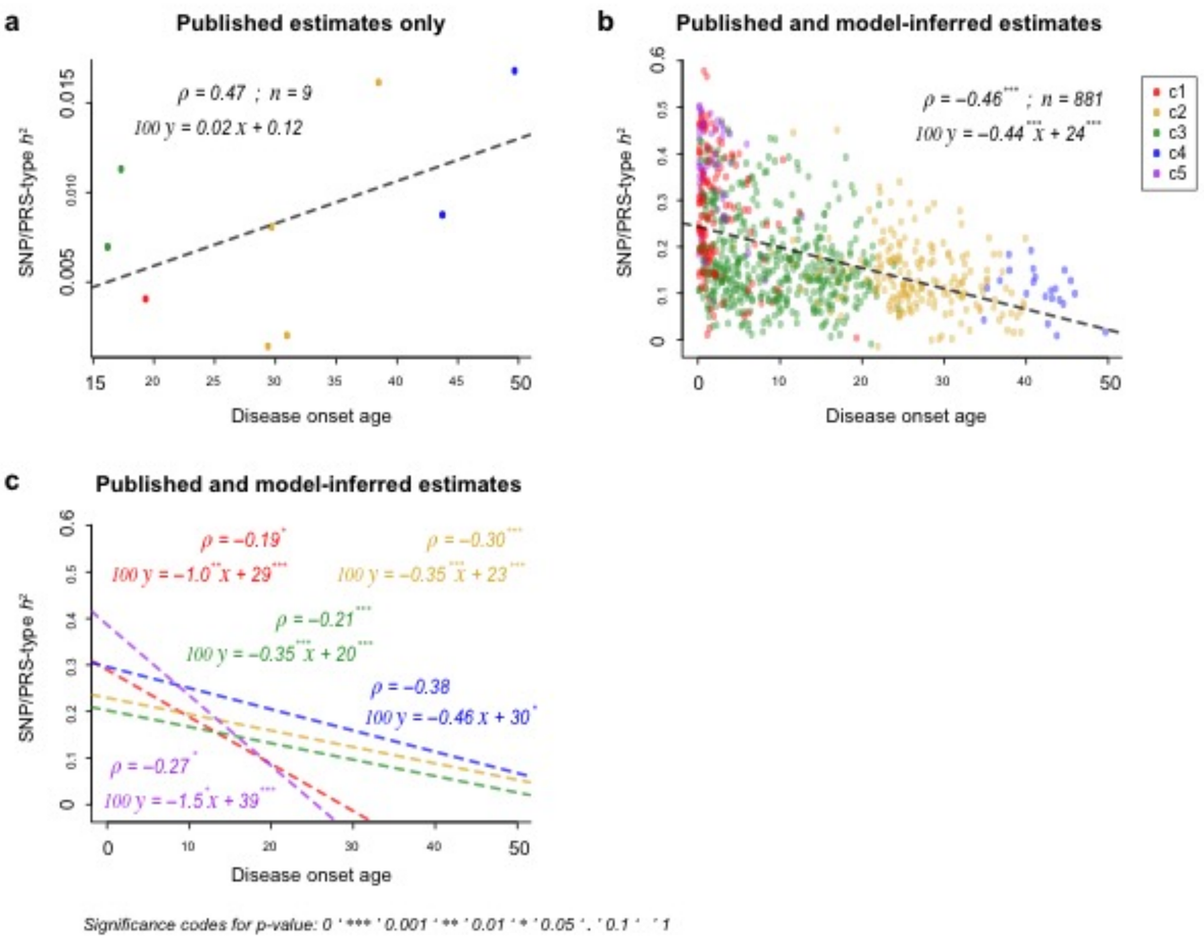

Supplementary Fig. 4 Relationships between disease onset age and SNP/PRS-based *h*² estimates (related to Fig. 4a-c).
We compared two types of h^2 estimates: One based on twin/family data (see Fig. 4a-c), and the other utilizing SNP/PRS data. Plate **a** includes analyses based on the previously-published estimates of SNP/PRS-type h^2 *only*, suggesting the scarcity of the legacy data. Plates **b** and **c** show analyses in which we also included new SNP/PRS-type estimates from our predictive model. Plate **b** shows that after we analyzed all five disease curve types jointly, we found a significantly negative correlation between disease onset age and the corresponding h^2 estimates. Plate **c** demonstrates the hidden heterogeneity across curve shape clusters underlying the overall linear relationship, which became apparent when we conducted the same analysis in a cluster-specific manner. For instance, the linear relationship in Cluster 1 (in red) is stronger than that in Clusters 2 and 3 (in yellow and green, respectively), showing a steeper slope of its best fitting line.

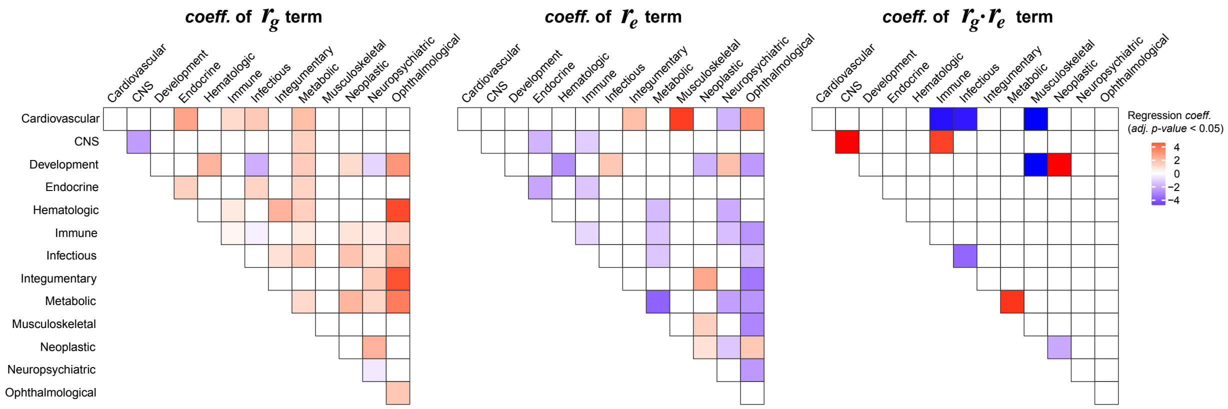

Supplementary Fig. 5 Regressing curve dissimilarity measure D_{soc} on estimates of r_g , r_e, and $r_g \cdot r_e$ (related to Supplementary Data 6)

Our analysis, described in this study, added hundreds of thousands of new r_g and r_e estimates. A simple D_{soc} regression on these correlation estimates in a disease-category-specific manner helped us interpret the relationship between inter-disease genetic and environmental correlations and the (dis)similarity of their prevalence curves. Similar to the analysis of the whole shape dissimilarities collection (described in the main text), we repeated the computation for all disease pairs sampled from distinct disease categories, generating three, upper triangular matrices of regression coefficients (for each type of correlation, and p-values were computed using Student's t test). The resulting plot shows only coefficients with Benjamini-Hochberg-adjusted p-values less than 0.05. We annotated the rows and columns with the 13 disease categories. The diagonal entries summarize the results for intra-category disease pairs and the off-diagonal for inter-category pairs.

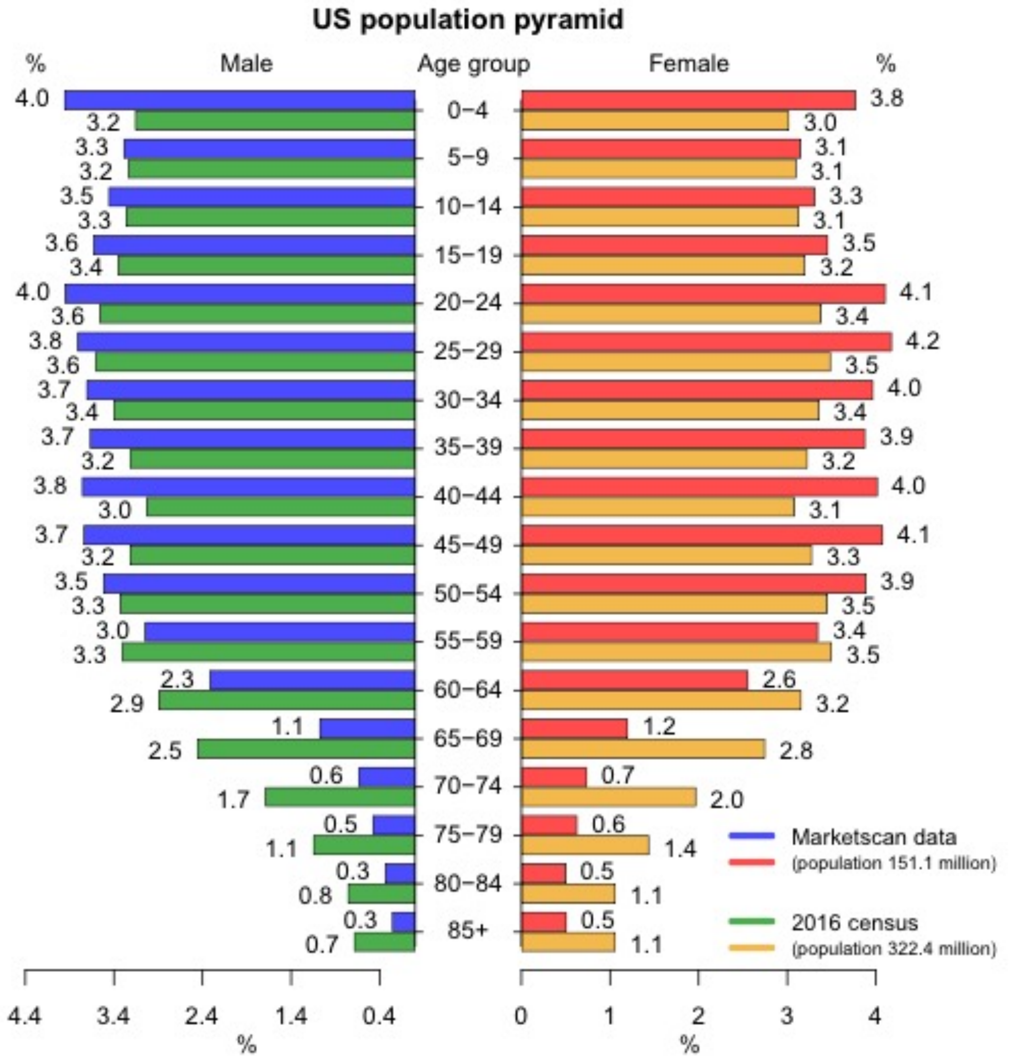

Supplementary Fig. 6 Population pyramid comparing sex-specific age group proportions between the MarketScan data and the 2016 US national census. The two datasets' pyramid shapes closely resemble each other, suggesting that the MarketScan database has a reasonable representation of all age groups, although the proportion younger than 54 years old is marginally larger. To generate this plot for the MarketScan dataset, we counted each individual only once, at their database entrance age (for the years from 2003 to 2013); this alleviates artifacts of variable patient visibility in the insurance claims.

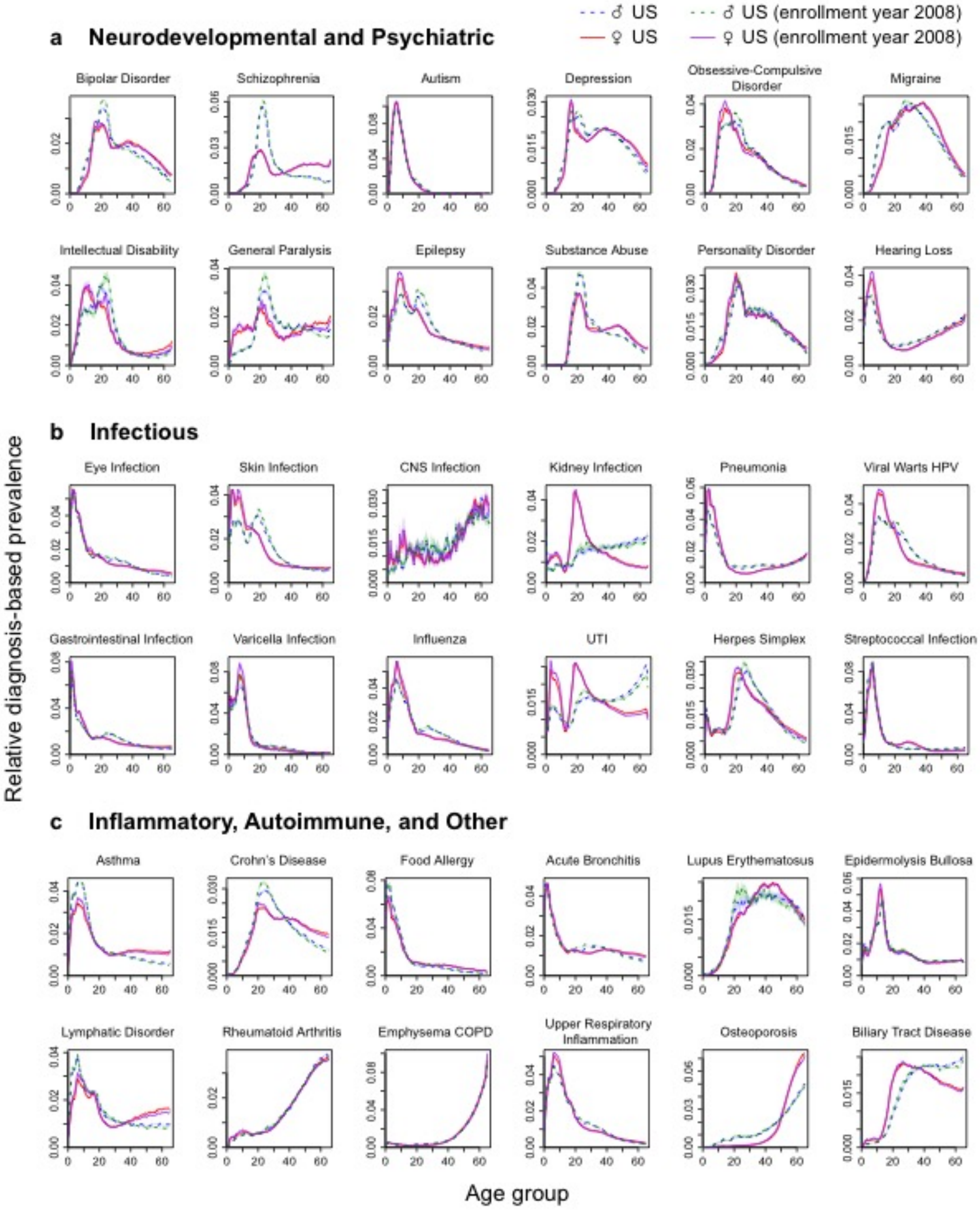

Supplementary Fig. 7 Disease curves for the year 2008's enrollment of several disease groups (related to Fig. 1a).

We computed the original curves based on enrollment years from 2003 to 2013 (the blue-dotted and red solid lines), and we compare these original curves with those constructed

using only one enrollment year, 2008, as an example (the green-dotted and purple solid lines). These old and new curves are practically identical, demonstrating the curve computation's robustness against the enrollment year. Three groups are shown, including **a** neurodevelopmental and psychiatric; **b** infectious, and; **c** inflammatory, autoimmune, and other. Each group here includes 12 diseases (labeled at the top). The *X*-axis corresponds to the age of diagnosis assignment, and the *Y*-axis shows the relative diagnosis-based prevalence (for ease of comparison of curves across countries, each curve is re-normalized to sum to 1). This also includes a 99 percent confidence interval for each curve.

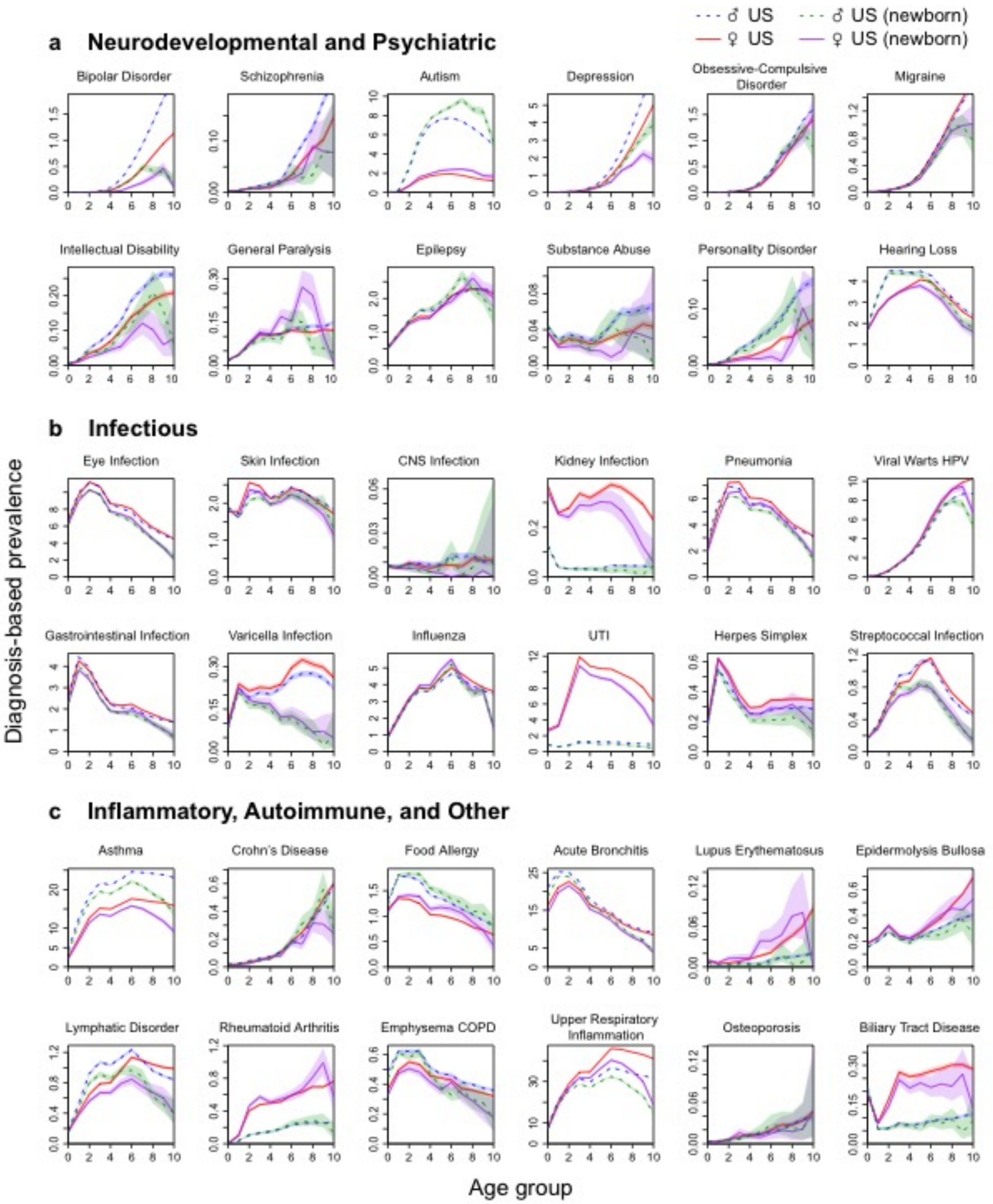

Supplementary Fig. 8 Disease curves of newborns for the same three groups of diseases as shown in Supplementary Fig. 7 (related to Fig. 1a).

We reproduced curves using only data for newborns visible in the MarketScan database (the green-dotted and purple solid lines). Same as Supplementary Fig. 7, each group

consists of 12 diseases (labeled at the top). The *X*-axis shows the diagnosis assignment age, and the *Y*-axis corresponds to the diagnosis-based prevalence (the expected number of diagnoses per 1,000 random samples). The 99 percent confidence interval is shown as a semi-transparent area in the matching color around each curve.

Supplementary Table 1. Feature Importance using Gradient Boosting Regression Method (related to Table 1).

Features derived from	95% CI of feature importance for h^2 prediction	95% CI of feature importance for *corr* prediction
**Curves**	44.6% $\pm$ 0.16%	41.2% $\pm$ 0.11%
**20 embedding factors (overall)**	36.8% $\pm$ 0.14%	30.7% $\pm$ 0.10%
**Individual breakdowns**		
**E1**	1.9%	2.0%
**E2**	2.2%	1.1%
**E3**	1.9%	1.6%
**E4**	2.0%	2.1%
**E5**	1.4%	1.5%
**E6**	1.7%	1.5%
**E7**	2.2%	0.8%
**E8**	2.6%	1.5%
**E9**	2.7%	1.5%
**E10**	1.5%	1.9%
**E11**	1.6%	0.3%
**E12**	1.4%	0.8%
**E13**	1.7%	0.7%
**E14**	1.5%	2.9%
**E15**	1.2%	1.6%
**E16**	1.9%	1.5%
**E17**	1.5%	2.5%
**E18**	1.7%	2.4%
**E19**	2.1%	1.8%
**E20**	2.0%	0.5%
**Data type and Math model**	8.3% $\pm$ 0.05%	9.0% $\pm$ 0.06%
**Country of cohort**	3.7% $\pm$ 0.03%	4.7% $\pm$ 0.05%
**Sex of cohort**	1.1% $\pm$ 0.02%	1.0% $\pm$ 0.02%
**Disease category**	1.1% $\pm$ 0.02%	1.1% $\pm$ 0.01%
**Disease onset age**	1.0% $\pm$ 0.02%	2.7% $\pm$ 0.03%

Supplementary Table 2. Correlation and Regression Analysis between Disease Onset Age and Diagnosis Count (related to Fig. 3f-g).

**Different stratifications**	**Spearman's ρ *(p-value)***	$log_{10}y = Ax + B$	
		A* *(p-value)	***B* *(p-value)***
**All**	0.32 ($< 10^{-16}$)	3.0×10^{-2} ($< 10^{-16}$)	5.1 ($< 10^{-16}$)
**Cluster 1**	9.7×10^{-2} (*ns* [a])	7.4×10^{-2} (1.3×10^{-2})	4.8 ($< 10^{-16}$)
**Cluster 2**	0.28 (5.2×10^{-5})	4.8×10^{-2} (3.4×10^{-7})	4.5 ($< 10^{-16}$)
**Cluster 3**	0.16 (1.6×10^{-4})	2.6×10^{-2} (2.8×10^{-4})	5.3 ($< 10^{-16}$)
**Cluster 4**	0.51 (1.6×10^{-2})	9.2×10^{-2} (7.4×10^{-3})	2.2 (*ns*)
**Cluster 5**	0.22 (*ns*)	0.17 (6.9×10^{-3})	4.4 ($< 10^{-16}$)

[a] *ns* : *p-value* > 0.05

Supplementary Table 3. Correlation and Regression Analysis between Disease Onset Age and Published h^2 without or with Model-inferred Values (related to Fig. 4a-c and Supplementary Fig. 4).

**Different strat.**	**Published h^2 only**								**Published and model-inferred h^2**							
	**Twin/Family-type**				**SNP/PRS-type**				**Twin/Family-type**				**SNP/PRS-type**			
	**# values**	**Spearman's ρ (p-value)**	**$100y = Ax + B$**		**# values**	**Spearman's ρ (p-value)**	**$100y = Ax + B$**		**# values**	**Spearman's ρ (p-value)**	**$100y = Ax + B$**		**# values**	**Spearman's ρ (p-value)**	**$100y = Ax + B$**	
			**A (p-value)**	**B (p-value)**			**A (p-value)**	**B (p-value)**			**A (p-value)**	**B (p-value)**			**A (p-value)**	**B (p-value)**
**All**	93	-0.44 (1.2×10^{-5})	-0.64 (1.2×10^{-4})	57 ($< 10^{-16}$)	9	0.47 (ns [a])	2.4×10^{-2} (ns)	0.12 (ns)	884	-0.46 ($< 10^{-16}$)	-0.42 ($< 10^{-16}$)	51 ($< 10^{-16}$)	881	-0.46 ($< 10^{-16}$)	-0.44 ($< 10^{-16}$)	24 ($< 10^{-16}$)
**Cluster 1**	11	-0.24 (ns)	-1.4 (ns)	67 (1.1×10^{-4})	1	–	–	–	152	-0.11 (ns)	-0.69 (1.6×10^{-2})	54 ($< 10^{-16}$)	152	-0.19 (2.1×10^{-2})	-1.0 (1.5×10^{-3})	29 ($< 10^{-16}$)
**Cluster 2**	18	-3.5×10^{-2} (ns)	25 (ns)	3081 (ns)	4	0.80 (ns)	0.14 (ns)	-3.8 (ns)	194	-0.25 (3.8×10^{-4})	-0.36 (1.7×10^{-4})	50 ($< 10^{-16}$)	193	-0.30 (3.1×10^{-5})	-0.35 (1.3×10^{-5})	23 ($< 10^{-16}$)
**Cluster 3**	46	-0.43 (3.2×10^{-3})	-136 (2.7×10^{-3})	6312 (5.2×10^{-15})	2	–	–	–	461	-0.25 (8.4×10^{-8})	-0.38 (1.0×10^{-6})	48 ($< 10^{-16}$)	459	-0.21 (5.1×10^{-6})	-0.35 (7.8×10^{-7})	20 ($< 10^{-16}$)
**Cluster 4**	5	0.20 (ns)	0.71 (ns)	9.2 (ns)	2	–	–	–	22	0.23 (ns)	0.58 (ns)	12 (ns)	22	-0.38 (ns)	-0.46 (ns)	30 (2.1×10^{-2})
**Cluster 5**	13	0.15 (ns)	0.66 (ns)	57 (1.7×10^{-4})	0	–	–	–	55	-9.7×10^{-2} (ns)	-0.61 (ns)	62 ($< 10^{-16}$)	55	-0.27 (5.0×10^{-2})	-1.5 (1.2×10^{-2})	39 ($< 10^{-16}$)

[a] ns : $p\text{-}value > 0.05$

Supplementary Table 4. Selected List of Representative h^2 Estimation Studies Used for Model Training.

Authors, Year (Database)	Key Features	Data Type	Math Model	# Values Used in Model
Polderman et al., 2015 (MaTCH) [1]	Meta-analysis on 17,804 traits from 2,748 publications	Twin study	ACE	88
Cole et al., 2009 [2]	Hyperactivity/inattention and mood in 645 twin pairs aged from 5 to 17 years in UK	Twin study	AE, ACE	8
Muñoz et al., 2016 [3]	12 complex diseases study based on 1,555,906 individuals of white ancestry from UK Biobank	Family study	ACE	20
Czene et al., 2002 [4]	15 common cancers study using 9.6 million individuals in Sweden	Family study	ACE	14
Polubriaginof et al., 2018 (RIFTEHR) [5]	500 phenotypes study based on identified 7.4 million familial relationships in US database	Family study using EHRs	AE, ACE	256
Wang et al., 2017 [6]	149 diseases study using 128,989 families in US	Family study using EHRs	ACE	148
Canela-Xandri et al., 2018 [7]	Study of 118 non-binary and 599 binary traits using UK Biobank (408,455 participants with over 30 million imputed SNPs)	SNP-based	GREML	123
Abbott et al., 2017 [8]	Study of over 2,000 traits using about 0.5 million individuals in UK Biobank	SNP-based	LDSC	81
Porcu et al., 2013 [9]	Meta-analysis of thyroid-related traits through GWAS in 26,420 and 17,520 individuals	PRS-based	PRS	2
Berndt et al., 2013 [10]	Meta-analysis of anthropometric traits with two stages, including 168,267 and 109,703 individuals of European ancestry, respectively	PRS-based	PRS	2

1. Polderman, T.J. *et al.* Meta-analysis of the heritability of human traits based on fifty years of twin studies. *Nat Genet* **47**, 702-9 (2015).

2. Cole, J., Ball, H.A., Martin, N.C., Scourfield, J. & McGuffin, P. Genetic Overlap Between Measures of Hyperactivity/Inattention and Mood in Children and Adolescents. *Journal of the American Academy of Child and Adolescent Psychiatry* **48**, 1094-1101 (2009).
3. Munoz, M. *et al.* Evaluating the contribution of genetics and familial shared environment to common disease using the UK Biobank. *Nat Genet* **48**, 980-3 (2016).
4. Czene, K., Lichtenstein, P. & Hemminki, K. Environmental and heritable causes of cancer among 9.6 million individuals in the Swedish family-cancer database. *International Journal of Cancer* **99**, 260-266 (2002).
5. Polubriaginof, F.C.G. *et al.* Disease Heritability Inferred from Familial Relationships Reported in Medical Records. *Cell* **173**, 1692-+ (2018).
6. Wang, K., Gaitsch, H., Poon, H., Cox, N.J. & Rzhetsky, A. Classification of common human diseases derived from shared genetic and environmental determinants. *Nature Genetics* **49**, 1319-+ (2017).
7. Canela-Xandri, O., Rawlik, K. & Tenesa, A. An atlas of genetic associations in UK Biobank. *Nature Genetics* **50**, 1593-+ (2018).
8. Abbott, L. *et al.* Heritability of >2,000 traits & disorders in UK Biobank. (2017).
9. Porcu, E. *et al.* A Meta-Analysis of Thyroid-Related Traits Reveals Novel Loci and Gender-Specific Differences in the Regulation of Thyroid Function. *Plos Genetics* **9**(2013).
10. Berndt, S.I. *et al.* Genome-wide meta-analysis identifies 11 new loci for anthropometric traits and provides insights into genetic architecture. *Nature Genetics* **45**, 501-U69 (2013).

Names and Legends for other Supplementary Data files (uploaded as separate files) are as follows:

Supplementary Data 1. A list of previously-published and our model-predicted heritability values.

Supplementary Data 2. A list of previously-published and our model-predicted correlation values.

Supplementary Data 3. Comparison of heritability estimates from an independent study and from our model prediction.

Supplementary Data 4. Separate comparisons of heritability estimates for acute and chronic diseases, from an independent study and from our model prediction.

Supplementary Data 5. Comparison of genetic correlation estimates from an independent study and from our model prediction.

Supplementary Data 6. Multi-variable Regression Analysis between Shape-of-cure Dissimilarity (D_{soc}) and Correlation Estimates of r_g and r_e, as well as Their Interaction Term (related to Fig. 4d and Supplementary Fig. 5): $D_{soc} = A\, r_g + B\, r_e + C\, r_g \bullet r_e + D_o$

Supplementary Data 7. A List of Disease Phenotypes, Grouped into Disease Categories (Underlined Bold Texts) and Sorted in Alphabetical Order
