# Supplementary material for: Estimating heritability and genetic correlations from large health datasets in the absence of genetic data
Source: Nat Commun. 2019 Dec 3;10:5508. doi: 10.1038/s41467-019-13455-0 (PMC6890770; doi:10.1038/s41467-019-13455-0)
Supplement: Supplementary file 9 — Description of Additional Supplementary Files [file 41467_2019_13455_MOESM9_ESM.pdf]

Title: Supplementary Data 1.
Description: A list of previously-published and our model-predicted heritability values.

Title: Supplementary Data 2.
Description: A list of previously-published and our model-predicted correlation values.

Title: Supplementary Data 3.
Description: Comparison of heritability estimates from an independent study and from our model prediction.

Title: Supplementary Data 4.
Description: Separate comparisons of heritability estimates for acute and chronic diseases, from an independent study and from our model prediction.

Title: Supplementary Data 5.
Description: Comparison of genetic correlation estimates from an independent study and from our model prediction.

Title: Supplementary Data 6.
Description: Multi-variable Regression Analysis between Shape-of-cure Dissimilarity (D_{soc}) and Correlation Estimates of r_g and r_e, as well as Their Interaction Term (related to Fig. 4d and Supplementary Fig. 5): $D_{soc} = A\, r_g + B\, r_e + C\, r_g \cdot r_e + D_0$

Title: Supplementary Data 7.
Description: A List of Disease Phenotypes, Grouped into Disease Categories (Underlined Bold Texts) and Sorted in Alphabetical Order
